# Abscisic Acid Signaling and Abiotic Stress Tolerance in Plants: A Review on Current Knowledge and Future Prospects

**DOI:** 10.3389/fpls.2017.00161

**Published:** 2017-02-20

**Authors:** Kanchan Vishwakarma, Neha Upadhyay, Nitin Kumar, Gaurav Yadav, Jaspreet Singh, Rohit K. Mishra, Vivek Kumar, Rishi Verma, R. G. Upadhyay, Mayank Pandey, Shivesh Sharma

**Affiliations:** ^1^Department of Biotechnology, Motilal Nehru National Institute of TechnologyAllahabad, India; ^2^Centre for Medical Diagnostic and Research, Motilal Nehru National Institute of TechnologyAllahabad, India; ^3^Amity Institute of Microbial Technology, Amity UniversityNoida, India; ^4^V.C.S.G Uttarakhand University of Horticulture and ForestryRanichauri, India; ^5^Department of computer Science and Engineering, Motilal Nehru National Institute of TechnologyAllahabad, India

**Keywords:** abiotic stress, phytohormone, abscisic acid, drought, radiation

## Abstract

Abiotic stress is one of the severe stresses of environment that lowers the growth and yield of any crop even on irrigated land throughout the world. A major phytohormone abscisic acid (ABA) plays an essential part in acting toward varied range of stresses like heavy metal stress, drought, thermal or heat stress, high level of salinity, low temperature, and radiation stress. Its role is also elaborated in various developmental processes including seed germination, seed dormancy, and closure of stomata. ABA acts by modifying the expression level of gene and subsequent analysis of *cis*- and *trans*-acting regulatory elements of responsive promoters. It also interacts with the signaling molecules of processes involved in stress response and development of seeds. On the whole, the stress to a plant can be susceptible or tolerant by taking into account the coordinated activities of various stress-responsive genes. Numbers of transcription factor are involved in regulating the expression of ABA responsive genes by acting together with their respective *cis*-acting elements. Hence, for improvement in stress-tolerance capacity of plants, it is necessary to understand the mechanism behind it. On this ground, this article enlightens the importance and role of ABA signaling with regard to various stresses as well as regulation of ABA biosynthetic pathway along with the transcription factors for stress tolerance.

## Introduction

There are numerous processes that stimulate the development and growth of plants. Such processes are continuously governed by the hormones released by plants (known as phytohormones). Out of five characteristic phytohormones, one is ABA which helps in controlling many development and growth characteristics of plants such as leaf abscission, inhibition of fruit ripening, etc. ABA is commonly known as the “stress hormone” that responds to variety of environmental stresses including both biotic and abiotic stress (Zhang, 2014). In a review, Wani et al. (2016) critically elaborated the importance of all major phytohormones in plant growth and development as well as abiotic stress tolerance, besides mentioning their engineering for conferring abiotic stress tolerance in transgenic crops.

Abscisic acid (ABA) is a tiny molecule and classified as a sesquiterpene. It has a non-planar configuration and has multiple functional moieties. The synthesis of ABA takes place *de novo* during drying up process and its degradation occurs during rehydration following dehydration (Roychoudhury et al., 2013). It occurs in plant roots and terminal buds at the top of plant. The C-15 ABA skeleton is commonly found in biosynthetic precursors such as xanthoxin, abscisic aldehyde, and abscisic alcohol as well as oxidized catabolites including phaseic acid, 8′-hydroxy-ABA and dihydrophaseic acid. The level of ABA produced endogenously is elicited in plant system due to several stress signals. These may include the stimulation of genes that encode for enzymes that forms ABA from β-carotene (Roychoudhury and Basu, 2012).

Abscisic acid has key roles in numerous cell-based processes *viz.* development of seed, vegetative growth, seed under development and sprouting and reaction to ecological stress (Xiong and Zhu, 2003). ABA is stable under high temperatures so as to get dissolved in boiling water without undergoing degradation (Zhang, 2014). It performs number of functions at cell level such as controlling the production of enzymes required for cell protection from dehydration (Finkelstein and Gibson, 2002; Li et al., 2014), thermal stress and regulating other processes like transfer of water (Assmann, 2003; Parent et al., 2009) or metabolism of iron. Itai and Ben-zioni (1974) proposed that response of plants can be controlled in a general way with respect to variations in micro-climatic parameters *viz.* temperature, moisture, and radiation by incorporating changes in ABA content. Enhanced amounts of ABA may perform an essential function during cold resistance. Such rise in the levels of ABA was witnessed in bean plant exudates when exposed to short duration of heat stress (Itai and Ben-zioni, 1974). ABA has been involved in the process of stomata closure in those times when there is not much requirement of CO_2_ or in drought conditions when the plant is not able to bear more loss of water via transpiration. Hence, at an organ-level, ABA is well-known for its essential role on movement of stomata (Assmann, 2003; Christmann et al., 2007), on tissue hydraulic conductivity (Hose et al., 2000; Parent et al., 2009), and on the growth of root and shoot. At complete plant level, ABA is thought to be a potential candidate for communication between roots and shoot during stress related to water and salt, and interaction with some other plant derived signals, but it also interact with other plant signals concerned with organ-to-organ communication. A major function exhibited by ABA is the inhibition of germination of seed. It is thought to deter the process of seed germination immediately after placing in soil.

Even though ABA is found to be stable over a wide pH range, it is transformed into γ-lactone in strictly acidic environment such as formic acid–hydrochloric acid (Mallaby and Ryback, 1972). It acts as a weak acid because of the presence of carboxy-group in side chain. Hence, the lipophilic nature of ABA relies highly on pH and supports more lipophilicity at lesser pH values. There are also various internal and external signals (environmental parameters) that control the growth and development of plants. For instance, ABA content is found to increase in the plants deprived of minerals and nitrogen (Xiong and Zhu, 2003). The photosensitivity of ABA is majorly contributed to the ring enone and the dienoic acid side chain. ABA undergoes photo-isomerization at C2 double bond when irradiated with UV light at 365 nm to produce equal amounts of ABA and 2-*trans*-ABA (2E-ABA). This form of ABA, i.e. 2E-ABA, is biologically inactive (Todoroki et al., 2001).

## Abscisic Acid Biosynthesis

Abscisic acid is a type of metabolite known as isoprenoids, or terpenoids. Isopentenyl (IDP) is a five-carbon (C5) precursor molecule from which it is derived. Originally, it was assumed that all isoprenoids are synthesized from mevalonate (MVA) until recently a secondary pathway has been identified for the synthesis of IDP, initially, in certain eubacteria and finally in higher plants (Nambara and Marion-Poll, 2005). Various enzymes are involved which utilizes β-carotene to synthesize ABA. In 1960, ABA was isolated and identified from cotton balls. Many plant varieties are capable of producing mutant ABA. Identifying such mutants along with their physiochemical properties has improved our knowledge of the biosynthesis pathway in other plant species.

**FIGURE 1:**
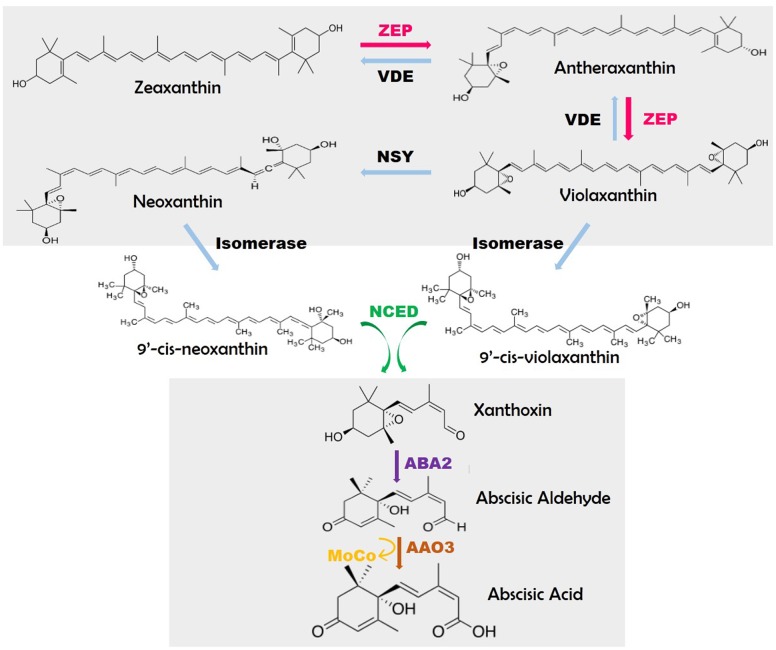
**Pathway of abscisic acid biosynthesis (modified from Nambara and Marion-Poll, 2005)**. Sources: **ZEP**: *Arabidopsis*-*Ataba1*/*npq2*/*los6, N. plumbaginifolia*-*Npaba2*, Rice-*Osaba1;* **NCED**: Maize-*vp14*, Tomato-*notabilis, Arabidopsis*-*Atnced3*; **ABA2**: *Arabidopsis*- *Ataba2/gin1/isi4/sis4;* **AAO3**: Tomato-*sitiens, Arabidopsis*-*aao3;* **MoCo**: Tomato-*flacca, N. plumbaginifolia-Npaba1, Arabidopsis*-*Ataba3/los5/gin5.*

Conversion of β-carotene to ABA is mediated via number of enzyme-catalyzed steps (**Figure 1**). The abiotic stress which entails triggering of assorted ABA bio-synthetic genes corresponding to zeaxanthin oxidase (ZEP), 9-*cis*-epoxycarotenoid dioxygenase (NCED), ABA-aldehyde oxidase (AAO) and molybdenum cofactor sulfurase (MCSU) might be because of calcium dependent phosphorylation pathway (Tuteja, 2007). Zeaxanthin is a trans-isomer form produced through cyclic hydroxylation of all-*trans*-lycopene via carotene. The initial step includes the synthesis of *cis*-isomers of violaxanthin and neoxanthin, each cleaved to generate C15 precursor of ABA (**Figure 1**). Regardless of the truth that ABA has 15 carbon atoms, it isn’t emanating immediately from the C15 sesquiterpene precursor, farnesyldiphosphate (FDP) in plants. However, it occurs via the cleavage of C40 carotenoids emerging from the MEP (2-C-methyl-d-erythritol-four- phosphate) pathway (Nambara and Marion-Poll, 2005).

Violaxanthin formation is catalyzed by zeaxanthinepoxidase (ZEP). This ZEP gene, firstly cloned in *Nicotiana plumbaginifolia* through insertional mutagenesis, codes for a protein having sequence resemblance to ferredoxin requiring FAD-binding monooxygenases (Nambara and Marion-Poll, 2005). These individual genes are triggered in seeds for ABA biosynthesis and deposition. In certain cases, the NtZEP expression surpasses the maximum limit within one-third to one-half of seed developmental stage and is linked with ABA accumulation during this period (Xiong and Zhu, 2003). In excessive mild stipulations, a reverse response happens in chloroplasts which are straight away catalyzed with the aid of violaxanthin de-epoxidase (VDE). The creation of *cis*-isomers of violaxanthin and neoxanthin could entail two enzymes, a neoxanthin synthase (NSY) and an isomerase. Cleavage of *cis*-xanthophylls is catalyzed by means of a family of 9-*cis*-epoxycarotenoid dioxygenases (NCED) (Bouvier et al., 1996). The ZmNCED gene was once remoted utilizing the maize vp14 mutant (Tan et al., 1997). Then xanthoxin is modified into abscisic aldehyde, by using a short-chain alcohol dehydrogenase (ABA2). The oxidation of the abscisic aldehyde into the carboxylic-acid is the last stage in ABA bio-synthesis, carried out by an abscisic aldehyde oxidase (AAO3). AAO3 protein comprises a molybdenum (Mo) cofactor activated through a MoCosulfurase.

Nine-*cis*-epoxycarotenoiddioxygenase (NCED) enzymes split the *cis*-isomers of violaxanthin and neoxanthin to generate C15 product, xanthoxin, and C-25 metabolite (**Figure 1**). The NCED gene (VP14) was first cloned in maize again with the help of insertional mutagenesis (Schwartz et al., 2003).

The biologically active form of ABA from *cis*-xanthoxin is executed involving two enzyme catalyzed steps with an intermediate abscisic aldehyde (**Figure 1**). Till date, the genes for those enzymes have been classified in *Arabidopsis* alone. The metamorphosis of xanthoxin to abscisic aldehyde is actuated by AtABA2 which belongs to the SDR family and is recognized by map-based cloning (Cheng et al., 2002; González-Guzmán et al., 2002) through isolation of numerous *Arabidopsis* mutant alleles from various genetic screens (González-Guzmán et al., 2002; Nambara and Marion-Poll, 2005).

Mutants lacking ABA plays a crucial role in displaying the pathway of ABA biosynthesis. Such mutants can be scrutinized by precocious germination of seeds and wilty appearance, such as in maize (*Zea mays*), tomato (*Lycopersicon esculentum*), tobacco (*Nicotiana tabacum*), potato (*Solanum tuberosum*), barley (*Hordeum vulgare*), and *Arabidopsis* (Zhang, 2014). Before the molecular roles of the impaired genes had been recognized, a common pathway for ABA biosynthesis was disclosed by profiling ABA biosynthetic intermediates and with feeding assays utilizing these mutants. The level of ABA in specific plant tissue is estimated by the rate of biosynthesis and catabolism activity of the hormone. Hence, identifying all the genes involved in biosynthesis improves our knowledge of how plant’s hormone directs growth and development.

### Regulation of ABA Biosynthesis through Abiotic Stress

The increase in *de novo* biosynthesis of ABA is due to the rise in abiotic stress which plays a role to inhibit its degradation and is thought to be stimulated by stress relief. Gene identified for ABA biosynthesis is ZEP and has been cloned and expressed in numerous plant species. This gene is found to be present in every plant part but is highly associated for basal expression in leaves (Xiong et al., 2002a). Moreover, level of ABA biosynthesis through ZEP gene is regulated not only in different plant portions and development phases but also in different plant species. The ZEP gene in *Arabidopsis* has a same basal transcript level in non-stressful conditions as in tobacco and tomato. These variations in the expression of ZEP genes are partly associated to basal transcript levels which also cover stress inducibility of genes as identified in different experiments. However, another ABA biosynthetic genes expression (NCED, AtAAO3, MCSU, and AtSDR1) are less debatable. The ABA biosynthesis is notably achieved after cleavage in the rate limiting step, and thus expression of NCED gene(s) has received a significant importance. NCED gene is found to be overexpressed in drought stress condition in maize (*Zea mays*; Tan et al., 1997), tomato (*Lycopersicon esculentum*; Burbidge et al., 1999), bean (*Phaseolus vulgaris*; Qin and Zeevaart, 1999), *Arabidopsis* (Iuchi et al., 2001), cowpea (*Vigna unguiculata*; Iuchi et al., 2000), and avocado (*Persea americana*; Chernys and Zeevaart, 2000). A remarkable rise in NCED transcript levels has been reported following 15–30 min of leaf extrication or induced dehydration (Xiong and Zhu, 2003), providing an evidence for the instant activation of NCED genes. Since ABA biosynthesis mechanism up-regulates drastically in response to stress, it can be deduced that protein levels of the related genes increases with the transcript levels, which were similarly noticed in NCED gene (Xiong and Zhu, 2003).

The end product ABA of the biosynthesis pathway negatively regulates the ABA accumulation via triggering its catabolic enzymes (Cutler and Krochko, 1999). The cytochrome P450 enzyme activity and ABA 8′- hydroxylase activity carries out the primary step of ABA degradation and was regulated by exogenous ABA accumulation. Since the product of NCED gene regulates the rate-limiting step in the ABA biosynthesis pathway, the information regarding the control of this gene product by ABA is very limited in terms of auto-regulation of ABA biosynthesis. In tomato plants and cowpea, this NCED gene was found to be not affected by exogenous ABA (Iuchi et al., 2000; Thompson et al., 2000). Hence, it can be concluded that ABA cannot stimulate its production but have the potential for its degradation.

The transcript measures 9-*cis*-epoxycarotenoid dioxygenase (NCED1) (an enzyme catalyzing the first step of ABA biosynthesis) and grows significantly in grape berries (Berli and Bottini, 2013). The resultant will be accumulation of ABA in different tissue pertaining to hormonal response ultimately causing a berry development and ripening (Wheeler et al., 2009).

However, Xiong and Zhu (2003) have shown that there is an up-regulation of ZEP, AAO3, and MCSU in *Arabidopsis* by ABA, even though being stimulated by stress. Exogenous ABA significantly regulates expression of such genes. Though, ABA biosynthesis production and degradation both are of greater significance in regulating ABA expression and adjusting plant stress responses and development strategies. As DRE- and ABRE-like *cis* elements are promoters of stress-inducible ABA genes (Xiong et al., 2001; Bray, 2002), consequently, these genes are in the same way controlled as DRE/CRT class of stress-responsive genes (Xiong et al., 2002b). Also, it has been shown that ABA induces second messengers activating defensive responses through the production of ROS (Sakamoto et al., 2008). Moreover, the expression of antioxidants enzyme gene and non-enzymatic defense systems genes are also activated by ABA signal induction mechanism (Jiang and Zhang, 2002).

With the help of ABA in stress response condition, screening can be done in vegetative tissues and would probably help to identify new loci which can be essential for regulating ABA metabolism (Zhang, 2014). ABA does specific types of acts which contain complicated regulatory mechanisms, production, degradation, signal perception, and transduction. Figuring out the crucial position of ABA in response to plant stress and their regulatory systems will help to formulate real-time techniques to procreate or genetically regulate crops with expanded tolerance to adverse environment stipulations.

## ABA and Abiotic Stress Signaling

The plant hormone ABA is an essential tool for developmental mechanism and robust stress responses to environmental stimuli (**Table 1**). Plant encounters more than a few abiotic stresses which involves larger concentration of salt (salinity), extremities of temperature (low temperature, i.e., cold (chilling or freezing), normal temperature (warmth) and water scarcity (drought or dehydration) (Fujita et al., 2011). Plant makes use of ABA to imitate the impact of a stress situation and might modify ABA stages continuously based on altering physiological and environmental conditions corresponding to seed dormancy and delays in germination, progress of seeds, promoting of stomatal closure, embryo morphogenesis, production of storage proteins and lipids, leaf senescence as well as safety toward pathogens (Tuteja, 2007). Exposure to environmental stress, as in case of drought, can produce detrimental effects on the progress of plants and the production of vegetation (**Table 1**). Abiotic stress is a damaging stress for environment, which inhibits crop production and yields even on irrigated land worldwide (Mahajan and Tuteja, 2005). Overall, stress is a multifaceted phenomenon which occurs at the time of their development. Plant responses vary depending on degree of the stress and the plant metabolome activity. In view that extra abiotic stresses motives dehydration of the cells and osmotic imbalance, there may be some normal gene expression pattern in cold, drought, high salt, or ABA application suggesting that stress signals and ABA metabolism have usual factors within the signaling pathway which is able to be in contact with each other for cellular homeostasis (Tuteja, 2007). It has been explored in a review by Rizwan et al. (2017) that plant tolerance against metal stress; increased plant growth, biomass, photosynthetic pigments, and gas exchange characteristics can be enhanced by application of endogenous and exogenous amendments of ABA.

**Table 1:** Details of abscisic acid regulation by stress and their effects.

Stress	Species	Organ	ABA	Effects	Reference
Desiccation	Maize (M3)	–	Endogenous	–	Close et al., 1989
Drought	*Arabidopsis*	Tissues	High	Prevention of ethylene production	Bray, 2002
Cd^2+^	Rice (TNG67)	Seedling	Increase	Stimulation of OASTL (for cysteine production)	Hsu and Kao, 2003
Cd^2+^ and drought	*B. juncea*	–	Endogenous	Transcription of aquaporins, ABA- and drought-responsive genes	Fusco et al., 2005
Drought	*Arabidopsis*	Leaves	Increase	safeguard the plant against disease	Mohr and Cahill, 2003
UV B	*Arabidopsis*	Leaves	Synthesis	Positive effect of UV B on ABA	Rakitin et al., 2008
UV B	Maize *vp14* mutant	Leaves	Increase	H_2_O_2_and NO **ccc**	Tossi et al., 2009
UV B	Grapevine	–	High	Tolerance to UV B	Berli et al., 2010, 2011
Cd^2+^	*B. juncea*	–	Endogenous	Up-regulation of BjCdR15 and TGA3	Farinati et al., 2010

A transformation in hormonal level is discovered in regard to stress including a rise in leaf ABA and/or a decline in cytokinins. Multiplied response of leaf ABA leads to cell wall extensibility and, in some plants, roots hydraulic conductance, and tissue turgor (**Table 1**). Decrease will result in the less uptake of carbon, resulting in carbohydrates accumulation and halts photosynthesis to combat the plant’s minimal need for carbohydrate. ABA leads to lowering of stomatal conductance, feedback inhibition of photosynthesis with the aid of carbohydrate accumulation, and decline in concentrations of photosynthetic enzymes are the principal motive for photosynthesis arrest (Evans, 1989).

### Heavy Metal Stress

Heavy metals are the foremost pollutants of environment, i.e., soil and water. Levels of some heavy metals like Cd, Cu, Pb, Hg, and Cr are high in agricultural and other natural areas which are because of human activities (Choudhary et al., 2010). Toxicity caused by heavy metals is the major cause of abiotic stress which leads humans, plants and animals to dangerous health effects (Singh et al., 2015, 2017; Tripathi et al., 2016a, 2017c). They have high reactivity and because of this they adversely influence energy synthesis, growth and senescence processes.

It is seemingly true that numerous physiological and developmental events are influenced by ABA. ABA notably increases freezing, chilling, drought, and salt tolerance in numerous plant species (Rikin and Richmond, 1976; Hsu and Kao, 2003). Heavy metals like Cd, Ni, Zn, and Al (Fediuc et al., 2005) have been revealed to raise ABA portions in plants (**Table 1**). One of the utmost toxic heavy metals is cadmium (Cd), a divalent heavy metal cation (Tripathi et al., 2012). Leakage of cadmium (Cd) in water, air and soil generally come as a discharge from mining, burning, industries and waste seepage, and by fertilization in fields with sewage sludge and phosphate. It is easily taken up by flora, which leads to lethal symptom such as reduced growth (Chen and Kao, 1995). It was also observed that cadmium damages photosynthesis of plants (Siedlecka and Baszynski, 1993), it lower chlorophyll level (Larsson et al., 1998), and inhibits the opening of stoma (Zhang, 2014). Fediuc et al. (2005) verified that roots have accumulated the Cd-induced ABA but it was not seen in shoots of *Phragmites* and *Typha* plants (**Table 1**).

Hsu and Kao (2003) reported the mechanism of cadmium resistance of rice crop from Taiwan. Their studies showed that at high temperature (30/35°C) in rice seedlings, ABA is involved for Cd resistance (**Table 1**). This result relies on observations that (a) in comparison of the level of endogenous ABA in Cd-sensitive cultivar (TN1) and Cd-tolerant cultivar (TNG67) it was found with increased level in latter one; (b) there is an increase in Cd tolerance of TN1 when exogenous dose of ABA was given; (c) whereas fluridone application reduced level of ABA, as well as cadmium tolerance level of TNG 67 seedlings; and (d) the fluridone effect on cadmium toxicity of TNG 67 seedlings was backed by the re-application of ABA. These results have shown the relationship that regulation of endogenous level of ABA biosynthesis can reduce Cd uptake in rice seedlings (Hsu and Kao, 2008). In a study by Wang et al. (2016), exogenous ABA was applied using two *Solanum photeinocarpum* ecotypes (mining and farmland) and found an increase in the Cd content in both ecotypes. Though, the association between Cd and ABA is dependent on plant species, application of ABA exogenously can produce results different from ABA production under Cd treatment (**Table 1**). Further, Pompeu et al. (2016) have shown that the mechanisms through which ABA is interacting with Cd involve evident histological and biochemical alterations. They have also indicated that the stress response of Cd is facilitated by ABA in tomato (**Table 1**). In the work of Fediuc et al. (2005), it’s proven that ABA mediated the Cd-precipitated stimulation of O-acetylserine (thiol) lyase (OASTL), the enzyme responsible for cysteine biosynthesis.

In a study carried out by Fusco et al. (2005), the expression of BjCdR39 (an aldehyde dehydrogenase) and BjCdR55 (RNA binding protein) was analyzed in ABA signaling. The expression of both BjCdR39 and BjCdR55, stimulated by cadmium in *Brassica juncea*, supported the involvement of ABA signal-transduction component in already present cross-talk between the cadmium-stimulated and water stress-stimulated signaling. The BjCdR51 and BjCdR49 denoting aquaporins PIP1 and PIP2 was found to be transcribed in *B. juncea* when exposed to Cd stress for a day along with expression of BjCdR55 and BjCdR39 (ABA- and drought-responsive gene). The above observation gave an indication that water stress is imposed by Cd and that Cd and ABA show synergistic relationship. BjCdR15 is a putative ortholog to *Arabidopsis* TGA3. TGA transcription factors belong to the group of bZIP transcription factors which are found in all eukaryotes. TGA factors bind specifically to TGACGTCA. BjCdR15 from *B. juncea* is up-regulated in plants treated for 6 h with cadmium (Fusco et al., 2005). When ABA treatment is given to the plant, both BjCdR15 and TGA3 responded to the treatment. However, more sensitivity was shown by TGA3 than BjCdR15 to ABA (Farinati et al., 2010).

Another major heavy metal is copper. Food chain is continuously infiltrated by Cu^2+^ contamination through soil which is a major threat to human health and has become an important concern for environment sustainability (Chary et al., 2008). It is essential in low quantity for usual plant growth and development but it lead to phytotoxicity at high concentrations (Berenguer et al., 2008). Against various biotic and abiotic stresses, plants develop a strong protection mechanism which consists the action of antioxidative enzymes by phytohormones such as auxins (Park et al., 2007), cytokinins (Zhang and Ervin, 2008), ABA (Staneloni et al., 2008), ethylene, salicylates, jasmonic acid, brassinosteriods, and polyamines (Choudhary et al., 2010).

Oxidative stress in plant’s metabolic reactions can be caused by metal induced effects of both Cr^6+^and Cu^2+^ which ultimately results in discharge of oxidants and free radicals. It is also shown that whenever there is Cr^6+^and Cu^2+^ stress there is increase in the synthesis of exogenous or endogenous ABA level. It further shows that the participation of ABA (**Table 1**) in heavy metal stress tolerance (Choudhary et al., 2010). The application of silicon (Si) has been known to increase the plant’s tolerance capacity against abiotic stresses. In a study, Kim et al. (2014) showed that Si significantly improved the growth and biomass of rice (*Oryza sativa*) plants and reduced the toxic effects of Cd/Cu after different stress periods. It was found that reduction in uptake of metals led to modulation of the ABA phytohormone involved in response to stress.

Srivastava et al. (2015) have mentioned the three essential ABA-related genes which includes majorly ABA-induced PP2C1 (HAI1), ABA insensitive 1 (ABI1), and ABA interactive protein 2 (AIP2) in *B. juncea*. GPX is a redox-related gene that is up-regulated under arsenic and copper stress in response to ABA. Under copper stress, GPX6 form (glutathione peroxidase gene encoding isoforms in cytosol and mitochondria) was observed to be intensely up-regulated in *Arabidopsis* (Milla et al., 2003). In another study, GPX3 had double roles in plant, i.e., homeostasis of hydrogen peroxide and signal relaying in guard cells. In turn, this signal is responsible for regulating stomata according to ABA (Miao et al., 2006). Therefore under arsenic stress, over-expression of GPX6 and GPX3 at separate time durations controlled the level of ROS and opening of stomata.

### Drought Stress

Drought is one of the principal abiotic stresses that negatively influence the growth of plant and yield (Tripathi et al., 2016b). Greater than half of the terrestrial region, consisting of the widespread portion of arable land, is susceptible to drought (Kogan, 1997). ABA, a phytohormone involved in the regulation of abiotic stress pathways in plants, is responsible for the plant response toward stress situations and additionally involved in other developmental process for example seed dormancy (Cutler et al., 2010; Fujita et al., 2011, 2013, 2014). Drought conditions create osmotic stress in organisms, which eventually cause desiccation and resistance to water uptake in plants. During osmotic stress conditions, ABA accrues (**Table 1**) and acts as a controller in stress response and tolerance of plants (Yamaguchi-Shinozaki and Shinozaki, 2006; Nakashima and Yamaguchi-Shinozaki, 2013). ABA is known to positively affect stress tolerance following exogenous application or through overexpressing genes for its increased endogenous content in plants. In a recent study, it was seen that when ABA, γ-aminobutyric acid (GABA) and salicylic acid (SA) were applied exogenously, it effectively improved the drought-induced damages in creeping bentgrass (*Agrostis stolonifera*) by maintaining membrane stability and leaf water status (Li et al., 2016). After analyzing its metabolic profile, it was found that ABA, GABA, and SA have influenced the common metabolic pathways and also caused differential changes in metabolite accumulation under drought stress (Li et al., 2016).

Some of the work has provided an evidence that water scarcity can have an effect on the expression of core ABA signaling constituents, equivalent to ABA, PYR/PYL/RCAR ABA receptors, protein phosphatases 2C (PP2Cs), and subclass III SnRK2 protein kinases (Weiner et al., 2010). Osmotic stresses, including drought, cold, and high salinity can cause cellular dehydration at the time of seed perfection in vegetative development (Fujita et al., 2011), and hence results in hyper-activation of plant ABA metabolism (Nambara and Marion-Poll, 2005) and transport (Kuromori et al., 2010). It was thought that drought induced-stress consequently enforce the high ABA level in *Arabidopsis* leaves and safeguard the plant against disease symptoms associated with an avirulent strain of *Pseudomonas syringae* pv. tomato (Mohr and Cahill, 2003; Tossi et al., 2009). In proliferating tissues, amount of ABA is 40-fold higher when going through drought and salt stress (Zeevaart and Creelman, 1988). Mutants having non-functional ABA bio-synthesis are extra vulnerable to environment deviations when compared to transgenic plants which can induce much hormonal response and forecast higher tolerance against abiotic stress than the wild type (Iuchi et al., 2001; Qin and Zeevaart, 2002). Huang et al. (2016) studied that ramie BnbZIP3 gene was upregulated in the presence of drought, high saline and ABA in ramie. BnbZIP3 gene belongs to the group of bZIP transcription factors. This might be due to the reason that the promoter of BnbZIP3 may contain number of *cis*-acting elements that are involved in ABA signaling and different stress responses.

The prime ABA-facilitated signaling pathway which incorporates PYR/PYL/RCARs, PP2Cs, SnRK2s, and bZIP transcription factors are fortunately classified under *in vitro* condition (Fujita et al., 2014). In short, major ABA signaling components actively regulates both fast and slow ABA communication pathway to tackle dehydration. Previous work reported the relation between osmotic stress by high salt or drought and two cellular pathways; one is ABA-dependent and another one ABA-independent. Cold stress response persuade through altered gene expression via an ABA-independent pathway. ABA-dependent pathway depends upon the availability of *cis*-acting element called ABRE element (ABA-responsive element) (Tuteja, 2007). Gene related studies indicate a co-relation between ABA-dependent and ABA-independent pathways and a cross talk or association of involved molecules in the signaling pathway. Calcium acts as a secondary messenger in response to stress and a potential candidate to assist cross communication. An ample amount of research work has proved that ABA, drought, cold and high salt induces a sudden rise in calcium levels in plant cells (Mahajan and Tuteja, 2005; Tuteja, 2007).

### UV-B Stress

The solar electromagnetic spectrum having wavelength between 200 and 400 nm belongs to the category of Ultraviolet (UV) radiation. The UV radiation is characterized as having a shorter wavelength in comparison with the photosynthetically active radiation (PAR) which has a wavelength between the range of 400–700 nm which in some extent over imposes with that people understand as violet. According to international standardization, the ultra violet rays encompasses three different types of radiation viz., UV-C, UV-B, and UV-A. The UV-C rays have wavelength between 200 and 280 nm a portion of shorter wavelength and consequently emit high energy photons which was absorbed completely by the ozone layer and not able to reach the surface of Earth.

Plants are categorized as sessile organisms which are attached to one place and required sunlight for their growth and therefore, they are certainly exposed to UV rays which contain approximately 7% of the electromagnetic radiation emanated from the sun (Tossi et al., 2009). Most of the UV-B radiations are captivated in the ozone layer in stratospheric layer and the remaining radiations are transmitted to the Earth’s surface (Tossi et al., 2009). A high percentage of UV-B rays stimulate the reactive oxygen species (ROS) which instigates damage to biomolecules and destroys membrane integrity, cell morphology as well as plant physiology and consequently affects plant growth and development which may be visible in several plant species (Frohnmeyer and Staiger, 2003; Tripathi et al., 2017a,b,c).

Abscisic acid functions as the main stimulating means of stomata closure, but in addition it imparts an important role in plants for their adaptations to drought conditions and to UV-B radiations (Sangtarash et al., 2009). The reaction mechanism of ABA involved the inhibition of ethylene synthesis in plant and enhances plant growth (Zhang, 2014). Ethylene production in plants increases by ultraviolet radiations (UV-B) as well as in water deficient conditions (**Table 1**). The plant species that experiences the exposure of UV-B radiations were found to be more tolerant to drought conditions and therefore ABA works in many ways toward plant’s reaction to drought conditions (Li et al., 2014).

It was studied previously that exposure to UV-B radiations in *Arabidopsis* cause resistance to pathogen named *Hyaloperonospora parasitica* (Kunz et al., 2008). It was also reported that plant exposed to UV-B radiations when treated with ABA restrict the expression of plant defensin 1.2, a gene involved in defense mechanism and get up-regulated by UV-B (Mackerness et al., 2001). A novel *cis*-regulatory element, i.e., UVBoxANAC13 was found to be regulated by UV-B radiation but suppressed during other environmental stress situations (Safrany et al., 2008). A study of Tossi et al. (2009) in maize plant shows that their leaves cause an increase in the production of ABA when exposed to UV-B radiation, and the concentration of H_2_O_2_ and NO was also enhanced in this particular condition. In addition ABA is needed for nitric oxide involved diminution of the deleterious effects generated by UV-B (**Table 1**). This proves that the upsurge in amount of ABA is generally the quickest response involved in the signaling pathways which is affected by UV-B radiations in maize leaves (**Table 1**).

Several studies attempt to relate the interaction incorporating UV-B and ABA, but some of it concludes that the presence of ABA enhance the tolerance of grapevines to UV-B (Berli et al., 2010, 2011). The same effect was observed by Rakitin et al. (2008) in leaves of *Arabidopsis* creating a positive effect of UV-B on ABA synthesis in tissues during high exposure to UV-B. Whereas additional studies reported that the interaction between water stress conditions to UV-B radiation observes a less sensitivity to UV-B in various plant species during drought conditions (Berli and Bottini, 2013)

It was observed in previous studies that ABA defends maize leaves during exposure of UV-B irradiation. The outcomes were observed by utilizing the *vp14* maize mutant which is not active for ABA synthesis. The *VP14* gene transcribes a 9-*cis*-epoxycarotenoid dioxygenase (NECD) enzyme (Tan et al., 1997). NECD cleaves epoxycarotenoids which converts into xanthoxin, which was further modified by a short-chain dehydrogenase/reductase (SDR1) to form abscisic aldehyde which is then converted into ABA by enzyme aldehyde oxidase (AO). The relation of ABA to nitric oxide regulation is still an issue of discussion. It was previously reported that either ABA- or UV-B induced NO is largely formed by NOS-like activity (i.e., ABA or UV-B might show NOS like activity to synthesize NO) (Qu et al., 2006). Whereas, several other pharmacological data suggest that nitrate reductase (NR) is the chief source of nitric oxide in guard cells in reaction to ABA-mediated H_2_O_2_ synthesis (Bright et al., 2006).

### Water Stress

In terms of plant productivity, it is surely considered that water scarcity is a chief restraining factor for plant growth under field conditions since plants are exposed to varying levels of water stress daily. Water shortage destroys many plant capabilities similar to photosynthesis, transpiration, stomatal conductance, and metabolite accumulation (Sangtarash et al., 2009), and therefore results in extensive decrease in plant growth and productivity (Reddy et al., 2004). ABA, a growth hormone in plant serves, many physiological processes in plants. Plant responses to drought incorporate alterations in morphology and biochemistry leading to acclimation in non-severe circumstances, and cause harm to plant and plant ingredients, in extreme cases (Li et al., 2014). Since water-stressed vegetation have more concentration of ABA than the good-watered crops, Sangtarash et al. (2009) speculated that the consequences of ABA will be highly enhanced for the sufficiently watered plants than for the water-stressed plants (**Table 1**).

It was assumed for the past 25 years that an increasing concentration of ABA in drought resistant plants also restricts growth of the plant; predominantly inhibit shoot growth (Trewavas and Jones, 1991). Several research mentioning the relationship between the ABA concentration of plant tissue or xylem sap and growth inhibition recommended that the enhanced concentration of endogenous ABA in drought resistant plant was enough to support a portion of plant growth, not all the growth inhibition resulted from water stress (Sangtarash et al., 2009).

Numerous studies have shown that due to the ABA content the specific mRNA and proteins accumulated late during seed embryogenesis in different plant species (Mundy and Chua, 1988). Physiological studies have proved that the escalation in endogenous ABA content in plant tissue resulted either from high osmoticum, NaCl, or drying conditions in water stressed plants (Li et al., 2014). Due to this, proteins and nucleic acid gets accumulate and cause intra-cellular osmolarity or have various defensive functions.

It has also been reported that ABA potentially inhibits shoot growth in appropriately watered plants (**Table 1**). Nevertheless, solely a sequence of reports was found previously that shows ABA deficiency in plant under drought conditions causes enhancement of shoot growth which is also in consistent with the expectation that endogenous ABA accumulation is responsible for plant growth inhibition. It was reported that the rate of shoot elongation was high in ABA deficient maize seedlings (fluridone-treated or vp5 mutant) in comparison with the control (Sharp et al., 1994). A study by Bray (2002) illustrated that an excessive awareness of ABA is required to hinder an overload ethylene creation from tissues below water stress stipulations. As a result, ABA accumulation in the course of drought could result in retaining shoot progress as well as root development, instead of avoiding growth which is more likely believed (**Table 1**).

Abscisic acid is known to regulate the balance between intrinsic growth and environmental responses. *AtABCG25* acts as cell-membrane ABA transporter exporting ABA from cytoplasm to outside of the cells. The plants with over-expressed *AtABCG25* shows a reduced transpiration phenotype without any growth retardation. In a work by Kuromori et al. (2016), it was observed that *AtABCG25* over-expression stimulated a local ABA response in guard cells. Furthermore, it was seen that *AtABCG25* overexpression increased the drought tolerance, probably resulting from maintenance of water contents over the common threshold for survival after drought stress treatment (Kuromori et al., 2016).

## Abscisic Acid Regulation of Seed Germination and Root Growth

Abscisic acid is one of essential phytohormones that help in regulation of developmental as well as physiological events occurring in plants. Such events involve seed dormancy, seedling development and growth, limiting the responses of many abiotic stresses (Bray, 2002; Finkelstein and Gibson, 2002; Assmann, 2003; Chow and McCourt, 2004; Yamaguchi-Shinozaki and Shinozaki, 2006). There are number of studies showing the number of genes involved in aforementioned processes. For example, the genes abi1 and abi2 hinder number of ABA responses which involves inhibition of germination process of seed and growth of seedling and support stomatal closure and other genes such as abi3, abi4, and abi5 only display ABA insensitivity during seed germination and premature seedling development (Fujii et al., 2007).

Another important process in signaling is phosphorylation. The significance of phosphorylation has been specified with the proper analysis of triggering of ABA response elements, i.e., ABRE binding factors which is also known as ABFs/ABREs. ABFs are transcription factors referred to as basic leucine zipper-type (bZIP) transcription factor which are involved in signaling of ABA. The parameters encoded by these ABA-responsive genes include recognized defensive proteins, enzymes prerequisite for osmolyte production or various transcription factors responsible for regulating other alterations in gene expression (Bray, 2002; Zhu, 2002).

The stomatal responses to ABA are positively regulated by the protein kinases making an exception to normal procedure (Mustilli et al., 2002; Yoshida et al., 2002). But protein kinases which are activated by ABA have not been reported for positive regulation of ABA responses. Of the *Arabidopsis* SnRK2s, SnRK2.2, SnRK2.3, SnRK2.6, SnRK2.7, and SnRK2.8 have been shown to be actuated by ABA after expressing in *Arabidopsis* protoplasts (Boudsocq et al., 2004).

Although there are number of genes that are concerned with signaling of ABA, certain crucial components are still under investigation. The signaling process of ABA is considered as an extremely branched system. As it is explained earlier that SnRK2.6 has been involved in positive regulation of ABA signaling, however, it only perform its function in ABA responses of guard cells (Mustilli et al., 2002; Fujii et al., 2007). Also, ABI1 and ABI2 perform negative regulation of germination of seed, growth of seedling and closure of stomata (Fujii et al., 2007). This situation highlights the question that which protein kinase might be present in the positive regulation of ABA signaling in germination of seed and growth of seedling. Further, snrk2.2 snrk2.3 plants show insensitivity toward ABA during seed-germination and indicate that SnRK2.2 and SnRK2.3 are the protein kinases responsible for positively regulating the signaling during seed germination. SnRK2.2 and SnRK2.3 are also concerned in other roles in seed dormancy, inhibition of seedling development by ABA, pro-stocking and gene expression prompted by means of ABA. Many of the ABA-induced genes showing decreased ABA responsiveness in snrk2.2 snrk2.3 are thought to carry ABREs in their promoter region. Therefore, SnRK2.2 and SnRK2.3 probably influence the expression of those genes via phosphorylating one or more than one ABFs and thereby, influences binding of ABF to ABRE (Fujii et al., 2007).

The continued existence of the next generation critically depends on controlling the germination of seed and seedling growth. There are also critical checkpoints at the stage of transition from dormant state to germination and from germination till the growth (Kermode, 2005). It was reported in a study that rare earth elements (REEs) shows adverse biological impact on growth and yield of plant (Jianrong et al., 2014). Though, when these REEs are incorporated in phytohormone ABA signaling is not clear. Recently in a study, interaction of Lanthanum (La^3+^) occurred with ABA signal in growing root of *Arabidopsis* (Jianrong et al., 2014). The amount of ABA used also affected this process, i.e., when 1 μmol/L of ABA was incorporated, rate of seed germination was inhibited and root was subjected to elongation in *Arabidopsis*. But when 10 μmol/L of La^3+^ was introduced, the effects produced by ABA were rescued. Furthermore, root hair development was promoted by ABA whereas the same was inhibited by La^3+^. Moreover, some studies revealed that H_2_O_2_ formation that was induced by ABA was further inhibited by La^3+^ (Jianrong et al., 2014). Overall, the interaction of La^3+^ with ABA may show a close relation with H_2_O_2_ signal regulated by La^3+^ in root cells. La^3+^ might act together with ABA upstream of H_2_O_2_ formation.

Various genic studies upon ABA regulation of seed germination as well as gene expression have recognized different mutants of *Arabidopsis* having different sensitivities to ABA (Chen et al., 2008). One of the ABA-insensitive mutants, *abi5*, was discovered for its capability to develop under high levels of exogenous ABA. As discussed earlier, *ABI5* codes for a bZIP transcription factor which, if accumulated, causes the inhibition of seed germination and initial growth of seedling (Lopez-Molina et al., 2001). Also, ABI5 regulates the expression of ABA induced which is specific to seed and *AtEM* genes codes for class I late embryogenesis abundant (LEA) proteins that are essential for seed maturation (Carles et al., 2002).

## Conclusion and Future Prospects

It is obvious that ABA being a significant signaling compound can transduce the signal perceived in response to various abiotic environmental stresses. Though, it is known that genes related to ABA have biological significance in advancing stress resistance, there is still a gap in research for generating crops having significantly enhanced resistance to stress in fields. Due to this, some genes that are shown to be effective under greenhouse trials for stress tolerance need to be evaluated in the field before being inculcated in breeding programs. Further it is necessary to unveil the complicated mechanisms for generating stress tolerance in plants by adapting more detailed and integrated genome-wise studies in order to locate the key components of developmental processes mediated by ABA and develop tools for engineering and breeding stress tolerant plants. Additionally, it becomes essential to figure out the roles and importance of all ABA-responsive genes to achieve elaborated vision of complex feature of abiotic stresses. In future, examining the influence of ABA induced genes on stress tolerance under combination of multiple stress conditions will give the detailed insight on working of ABA.

## Author Contributions

KV, NU, NK, GY, JS, SS, VK, RU, and RM designed the manuscript. KV, NU, NK, GY, JS, RV, and RM wrote the manuscript. KV, NK, SS, MP, and RU critically evaluated the manuscript.

## Conflict of Interest Statement

The authors declare that the research was conducted in the absence of any commercial or financial relationships that could be construed as a potential conflict of interest.

## References

[B1] Assmann S. M. (2003). OPEN STOMATA1 opens the door to ABA signaling in *Arabidopsis* guard cells. *Trends Plant Sci.* 8 151–153. 10.1016/S1360-1385(03)00052-9 12711225

[B2] Berenguer P. Cela S. Santivera F. Boixadera J. Lloveras J. (2008). Copper and zinc soil accumulation and plant concentration in irrigated maize fertilized with liquid swine manure. *Agron. J.* 100 1056–1061. 10.2134/agronj2007.0321

[B3] Berli F. J. Bottini R. (2013). UV-B and abscisic acid effects on grape berry maturation and quality. *J. Berry Res.* 3 1–14.

[B4] Berli F. J. Fanzone M. Piccoli P. Bottini R. (2011). Solar UV-B and ABA are involved in phenol metabolism of *Vitis vinifera* L. increasing biosynthesis of berry skin polyphenols. *J. Agric. Food Chem.* 59 4874–4884. 10.1021/jf200040z 21469737

[B5] Berli F. J. Moreno D. Piccoli P. Hespanhol-Viana L. Silva M. F. Bressan-Smith R. (2010). Abscisic acid is involved in the response of grape (*Vitis vinifera* L.) cv. Malbec leaf tissues to ultraviolet-B radiation by enhancing ultraviolet- absorbing compounds, antioxidant enzymes and membrane sterols. *Plant Cell Environ.* 33 1–10. 10.1111/j.1365-3040.2009.02044.x 19781012

[B6] Boudsocq M. Barbier-Brygoo H. Lauriere C. (2004). Identification of nine sucrose nonfermenting 1-related protein kinases 2 activated by hyperosmotic and saline stresses in *Arabidopsis thaliana*. *J. Biol. Chem.* 279 41758–41766. 10.1074/jbc.M405259200 15292193

[B7] Bouvier F. d’Harlingue A. Hugueney P. Marin E. Marion-Poll A. Camara B. (1996). Xanthophyll biosynthesis. Cloning, expression, functional reconstitution, and regulation of β-cyclohexenyl carotenoid epoxidase from pepper (*Capsicum annuum*). *J. Biol. Chem.* 271 28861–28867. 8910532 10.1074/jbc.271.46.28861

[B8] Bray E. A. (2002). Abscisic acid regulation of gene expression during water deficit stress in the era of the *Arabidopsis* genome. *Plant Cell Environ.* 25 153–161. 10.1046/j.1365-3040.2002.00746.x 11841660

[B9] Bright J. Desikan R. Hancock J. T. Weir I. S. Neil S. J. (2006). ABA-induced NO generation and stomatal closure in *Arabidopsis* are dependent on H2O2 synthesis. *Plant J.* 45 113–122. 10.1111/j.1365-313X.2005.02615.x 16367958

[B10] Burbidge A. Grieve T. M. Jackson A. Thompson A. McCarty D. R. Taylor I. B. (1999). Characterization of the ABA-deficient tomato mutant *notabilis* and its relationship with maize *vp14*. *Plant J.* 17 427–431. 10.1046/j.1365-313X.1999.00386.x 10205899

[B11] Carles C. Bies-Etheve N. Aspart L. Léon-Kloosterzie K. M. Koornneef M. Echeverria M. (2002). Regulation of *Arabidopsis thaliana* EM genes: Role of ABI5. *Plant J.* 30 373–383. 10.1046/j.1365-313X.2002.01295.x 12000684

[B12] Chary N. S. Kamala C. T. Raj D. S. (2008). Assessing risk of heavy metals from consuming food grown on sewage irrigated soils and food chain transfer. *Ecotoxicol. Environ. Saf.* 69 513–524. 10.1016/j.ecoenv.2007.04.013 17555815

[B13] Chen H. Zhang J. Neff M. M. Hong S. W. Zhang H. Deng X. W. (2008). Integration of light and abscisic acid signaling during seed germination and early seedling development. *Proc. Natl. Acad. Sci. U.S.A.* 105 4495–4500. 10.1073/pnas.0710778105 18332440 PMC2393781

[B14] Chen S. L. Kao C. H. (1995). Cd induced changes in proline level and peroxidase activity in roots of rice seedlings. *Plant Growth Regul.* 17 67–71. 10.1007/BF00024497

[B15] Cheng W. H. Endo A. Zhou L. Penney J. Chen H. C. Arroyo A. (2002). A unique short-chain dehydrogenase/reductase in *Arabidopsis* glucose signaling and abscisic acid biosynthesis and functions. *Plant Cell* 14 2723–2743. 10.1105/tpc.006494 12417697 PMC152723

[B16] Chernys J. T. Zeevaart J. A. (2000). Characterization of the 9-cisepoxycarotenoid dioxygenase gene family and the regulation of abscisic acid biosynthesis in avocado. *Plant Physiol.* 124 343–353. 10.1104/pp.124.1.343 10982448 PMC59148

[B17] Choudhary S. P. Bhardwaj R. Gupta B. D. Dutt P. Gupta R. K. Kanwar M. (2010). Changes induced by Cu2+ and Cr6+ metal stress in polyamines, auxins, abscisic acid titers and antioxidative enzymes activities of radish seedlings. *Braz. J. Plant Physiol.* 22 263–270. 10.1590/S1677-04202010000400006

[B18] Chow B. McCourt P. (2004). Hormone signaling from a developmental context. *J. Exp. Bot.* 55 247–251. 10.1093/jxb/erh032 14673027

[B19] Christmann A. Weiler E. W. Steudle E. Grill E. (2007). A hydraulic signal in root-to-shoot signaling of water shortage. *Plant J.* 52 167–174. 10.1111/j.1365-313X.2007.03234.x 17711416

[B20] Close T. J. Kortt A. A. Chandler P. M. (1989). A cDNA-based comparison of dehydration-induced proteins (dehydrins) in barley and corn. *Plant Mol. Biol.* 13 95–108. 10.1007/BF00027338 2562763

[B21] Cutler A. Krochko J. (1999). Formation and breakdown of ABA. *Trends Plant Sci.* 4 472–478. 10.1016/S1360-1385(99)01497-1 10562731

[B22] Cutler S. R. Rodriguez P. L. Finkelstein R. R. Abrams S. R. (2010). Abscisic acid: emergence of a core signaling network. *Annu. Rev. Plant Biol.* 61 651–679. 10.1146/annurev-arplant-042809-112122 20192755

[B23] Evans. (1989). Photosynthesis and nitrogen relationships in leaves of C3 plants. *Oecologia (Berlin)* 78 9–19. 10.1007/BF00377192 28311896

[B24] Farinati S. DalCorso G. Varotto S. Furini A. (2010). The *Brassica juncea* BjCdR15 an ortholog of *Arabidopsis* TGA3 is a regulator of cadmium uptake, transport and accumulation in shoots and confers cadmium tolerance in transgenic plants. *New Phytol.* 185 964–978. 10.1111/j.1469-8137.2009.03132.x 20028476

[B25] Fediuc E. Lips S. H. Erdei L. (2005). O-Acetylserine (thiol) lyase activity in *Phragmites* and *Typha* plants under cadmium and NaCl stress conditions and the involvement of ABA in the stress response. *J. Plant Physiol.* 162 865–872. 10.1016/j.jplph.2004.11.015 16146312

[B26] Finkelstein R. R. Gibson S. I. (2002). ABA and sugar interactions regulating development. Cross-talk or voices in a crowd? *Curr. Opin. Plant Biol.* 5 26–32. 10.1016/S1369-5266(01)00225-4 11788304

[B27] Frohnmeyer H. Staiger D. (2003). Ultraviolet-B radiation-mediated responses in plants. Balancing damage and protection. *Plant Physiol.* 133 1420–1428. 10.1104/pp.103.030049 14681524 PMC1540342

[B28] Fujii H. Verslues P. E. Zhu J. K. (2007). Identification of two protein kinases required for abscisic acid regulation of seed germination, root growth, and gene expression in *Arabidopsis*. *Plant Cell* 19 485–494. 10.1105/tpc.106.048538 17307925 PMC1867333

[B29] Fujita Y. Fujita M. Shinozaki K. Yamaguchi-Shinozaki K. (2011). ABA-mediated transcriptional regulation in response to osmotic stress in plants. *J. Plant Res.* 124 509–525. 10.1007/s10265-011-0412-3 21416314

[B30] Fujita Y. Nakashima K. Yoshida T. Fujita M. Shinozaki K. Yamaguchi-Shinozaki K. (2014). “Role of abscisic acid signaling in drought tolerance and pre-harvest sprouting under climate change,” in *Climate Change and Plant Abiotic Stress Tolerance*, eds Tuteja N. Gill S. S. (Weinheim: Wiley-VCH Verlag GmbH and Co. KGaA), 521–553.

[B31] Fujita Y. Yoshida T. Yamaguchi- Shinozaki K. (2013). Pivotal role of the AREB/ABF–SnRK2 pathway in ABRE mediated transcription in response to osmotic stress in plants. *Physiol. Plant.* 147 15–27. 10.1111/j.1399-3054.2012.01635.x 22519646

[B32] Fusco N. Micheletto L. DalCorso G. Borgato L. Furini A. (2005). Identification of cadmium-regulated genes by cDNA-AFLP in the heavy metal accumulator *Brassica juncea* L. *J. Exp. Bot.* 56 3017–3027. 10.1093/jxb/eri299 16216843

[B33] González-Guzmán M. Apostolova N. Bellés J. M. Barrero J. M. Piqueras P. Ponce M. R. (2002). The short-chain alcohol dehydrogenase ABA2 catalyzes the conversion of xanthoxin to abscisic aldehyde. *Plant Cell* 14 1833–1846. 10.1105/tpc.002477 12172025 PMC151468

[B34] Hose E. Steudle E. Hartung W. (2000). Abscisic acid and hydraulic conductivity of maize roots: a study using cell- and root-pressure probes. *Planta* 211 874–882. 10.1007/s004250000412 11144273

[B35] Hsu Y. T. Kao C. H. (2003). Role of abscisic acid in cadmium tolerance of rice (*Oryza sativa* L.) seedlings. *Plant Cell Environ.* 26 867–874. 10.1046/j.1365-3040.2003.01018.x 12803614

[B36] Hsu Y. T. Kao C. H. (2008). Distinct roles of abscisic acid in rice seedlings during cadmium stress at high temperature. *Bot. Stud.* 49 335–342.

[B37] Huang C. Zhou J. Jie Y. Xing H. Zhong Y. She W. (2016). A ramie (*Boehmeria nivea*) bZIP transcription factor BnbZIP3 positively regulates drought, salinity and heavy metal tolerance. *Mol. Breed.* 36:120 10.1007/s11032-016-0470-2

[B38] Itai C. Ben-zioni A. (1974). “Regulation of plant response to high temperature,” in *Mechanisms of Regulation of Plant Growth*, eds Bieleski R. L. Ferguson A. R. Cresswell M. M. (Wellington: The Royal Society of New Zealand), 477–482.

[B39] Iuchi S. Kobayashi M. Taji T. Naramoto M. Seki M. Kato T. (2001). Regulation of drought tolerance by gene manipulation of 9-cis-epoxycarotenoid dioxygenase, a key enzyme in abscisic acid biosynthesis in *Arabidopsis*. *Plant J.* 27 325–333. 10.1046/j.1365-313x.2001.01096.x 11532178

[B40] Iuchi S. Kobayashi M. Yamaguchi-Shinozaki K. Shinozaki K. (2000). A stress-inducible gene for 9-cis-epoxycarotenoid dioxygenase involved in abscisic acid biosynthesis under water stress in drought-tolerant cowpea. *Plant Physiol.* 123 553–562. 10.1104/pp.123.2.553 10859185 PMC59023

[B41] Jiang M. Zhang J. (2002). Role of abscisic acid in water stress-induced antioxidant defense in leaves of maize seedlings. *Free Radic. Res.* 36 1001–1015. 10.1080/1071576021000006563 12448826

[B42] Jianrong W. Lei W. Ting H. Wenchao L. Shaowu X. (2014). Effects of lanthanum on abscisic acid regulation of root growth in *Arabidopsis*. *J. Rare Earths* 32 78–82. 10.1016/S1002-0721(14)60035-1

[B43] Kermode A. R. (2005). Role of abscisic acid in seed dormancy. *J. Plant Growth Regul.* 24 319–344. 10.1007/s00344-005-0110-2

[B44] Kim Y. H. Khan A. L. Kim D. H. Lee S. Y. Kim K. M. Waqas M. (2014). Silicon mitigates heavy metal stress by regulating P-type heavy metal ATPases, *Oryza sativa* low silicon genes, and endogenous phytohormones. *BMC Plant Biol.* 14:13 10.1186/1471-2229-14-13 PMC3893592 24405887

[B45] Kogan F. N. (1997). Global drought watch from space. *Bull. Am. Meteorol. Soc.* 78 621–636. 10.1175/1520-0477(1997)078<0621:GDWFS>2.0.CO;2

[B46] Kunz B. A. Dando P. K. Grice D. M. Mohr P. G. Schenk P. M. Cahill D. M. (2008). UV induced DNA damage promotes resistance to the biotrophic pathogen *Hyaloperonospora parasitica* in *Arabidopsis*. *Plant Physiol.* 148 1021–1031. 10.1104/pp.108.125435 18667719 PMC2556815

[B47] Kuromori T. Fujita M. Urano K. Tanabata T. Sugimoto E. Shinozaki K. (2016). Overexpression of AtABCG25 enhances the abscisic acid signal in guard cells and improves plant water use efficiency. *Plant Sci.* 251 75–81. 10.1016/j.plantsci.2016.02.019 27593465

[B48] Kuromori T. Miyaji T. Yabuuchi H. Shimizu H. Sugimoto E. Kamiya A. (2010). ABC transporter AtABCG25 is involved in abscisic acid transport and responses. *Proc. Natl. Acad. Sci. U.S.A.* 107 2361–2366. 10.1073/pnas.0912516107 20133881 PMC2836683

[B49] Larsson E. H. Bordman J. F. Asp H. (1998). Influence of UV-B radiation and Cd2+ on chlorophyll fluorescence, growth and nutrient content in *Brassica napus*. *J. Exp. Bot.* 49 1031–1039. 10.1093/jxb/49.323.1031

[B50] Li C. Yue J. Wu X. Xu C. Yu J. (2014). An ABA-responsive DRE-binding protein gene from *Setaria italica*, SiARDP, the target gene of SiAREB, plays a critical role under drought stress. *J. Exp. Bot.* 65 5415–5427. 10.1093/jxb/eru302 25071221 PMC4157718

[B51] Li Z. Yu J. Peng Y. Huang B. (2016). Metabolic pathways regulated by abscisic acid, salicylic acid and γ-aminobutyric acid in association with improved drought tolerance in creeping bentgrass (*Agrostis stolonifera*). *Physiol. Plant.* 159 42–58. 10.1111/ppl.12483 27507681

[B52] Lopez-Molina L. Mongrand S. Chua N. H. (2001). A post germination developmental arrest checkpoint is mediated by abscisic acid and requires the ABI5 transcription factor in *Arabidopsis*. *Proc. Natl. Acad. Sci. U.S.A.* 98 4782–4787. 10.1073/pnas.081594298 11287670 PMC31911

[B53] Mackerness S.A-H. John C. F. Jordan B. Thomas B. (2001). Early signaling components in ultraviolet-B responses: distinct roles for different reactive oxygen species and nitric oxide. *FEBS Lett.* 489 237–242. 10.1016/S0014-5793(01)02103-2 11165257

[B54] Mahajan S. Tuteja N. (2005). Cold, salinity and drought stresses: an overview. *Arch. Biochem. Biophys.* 444 139–158. 10.1016/j.abb.2005.10.018 16309626

[B55] Mallaby R. Ryback G. (1972). Chemistry of a color test for abscisic acid. *J. Chem. Soc.* 8 919–921. 10.1016/j.jplph.2014.07.009

[B56] Miao Y. Lv D. Wang P. Wang X. C. Chen J. Miao C. (2006). An *Arabidopsis* glutathione peroxidase functions as both a redox transducer and a scavenger in abscisic acid and drought stress responses. *Plant Cell* 18 2749–2766. 10.1105/tpc.106.044230 16998070 PMC1626619

[B57] Milla M. A. R. Maurer A. Rodriguez Huete A. Gustafson J. P. (2003). Glutathione peroxidase genes in *Arabidopsis* are ubiquitous and regulated by abiotic stresses through diverse signaling pathways. *Plant J.* 36 602–615. 10.1046/j.1365-313X.2003.01901.x 14617062

[B58] Mohr P. G. Cahill D. M. (2003). Abscisic acid influences the susceptibility of *Arabidopsis thaliana* to *Pseudomonas syringae* pv. tomato and *Peronospora parasitica*. *Funct. Plant Biol.* 30 461–469. 10.1071/FP02231 32689031

[B59] Mundy J. Chua N. (1988). Abscisic acid and water-stress induce the expression of a novel rice gene. *EMBO J.* 7 2279–2286. 2973410 10.1002/j.1460-2075.1988.tb03070.x PMC457090

[B60] Mustilli A. C. Merlot S. Vavasseur A. Fenzi F. Giraudat J. (2002). *Arabidopsis* OST1 protein kinase mediates the regulation of stomatal aperture by abscisic acid and acts upstream of reactive oxygen species production. *Plant Cell* 14 3089–3099. 10.1105/tpc.007906 12468729 PMC151204

[B61] Nakashima K. Yamaguchi-Shinozaki K. (2013). ABA signaling in stress-response and seed development. *Plant Cell Rep.* 32 959–970. 10.1007/s00299-013-1418-1 23535869

[B62] Nambara E. Marion-Poll A. (2005). Abscisic acid biosynthesis and catabolism. *Annu. Rev. Plant Biol.* 56 165–185. 10.1146/annurev.arplant.56.032604.144046 15862093

[B63] Parent B. Hachez C. Redondo E. Simonneau T. Chaumont F. Tardieu F. (2009). Drought and abscisic acid effects on aquaporin content translate into changes in hydraulic conductivity and leaf growth rate: a trans-scale approach. *Plant Physiol.* 149 2000–2012. 10.1104/pp.108.130682 19211703 PMC2663730

[B64] Park J. E. Park J. Y. Kin Y. S. Staswick P. E. Jeon J. Yun J. (2007). GH3-medaited auxin homeostasis links growth regulation with stress adaptation response in *Arabidopsis*. *J. Biol. Chem.* 282 10036–10046. 10.1074/jbc.M610524200 17276977

[B65] Pompeu G. B. Vilhena M. B. Gratão P. L. Carvalho R. F. Rossi M. L. Martinelli A. P. (2016). Abscisic acid-deficient sit tomato mutant responses to cadmium-induced stress. *Protoplasma* 10.1007/s00709-016-0989-4 [Epub ahead of print] 27263082

[B66] Qin X. Zeevaart J. A. (2002). Overexpression of a 9-cisepoxycarotenoid dioxygenase gene in *Nicotiana plumbaginifolia* increases abscisic acid and phaseic acid levels and enhances drought tolerance. *Plant Physiol.* 128 544–551. 10.1104/pp.010663 11842158 PMC148917

[B67] Qin X. Zeevaart J. A. D. (1999). The 9-cis-epoxycarotenoid cleavage reaction is the key regulatory step of abscisic acid biosynthesis in water-stressed bean. *Proc. Natl. Acad. Sci. U.S.A.* 96 15354–15361. 10.1073/pnas.96.26.15354 10611388 PMC24823

[B68] Qu Y. Feng H. Wang Y. Zhang M. Cheng J. Wang X. (2006). Nitric oxide functions as a signal in ultraviolet-B induced inhibition of pea stems elongation. *Plant Sci.* 170 994–1000. 10.1016/j.plantsci.2006.01.003

[B69] Rakitin V. Y. Karyagin V. V. Rakitina T. Y. Prudnikova O. N. Vlasov P. V. (2008). UV-B stress-induced ABA production in *Arabidopsis thaliana* mutants defective in ethylene signal transduction pathway. *Russ. J. Plant Physiol.* 55 854–856. 10.1134/S1021443708060174

[B70] Reddy A. R. Chaitanya K. V. Vivekanandan M. (2004). Drought-induced responses of photosynthesis and antioxidant metabolism in higher plants. *J. Plant Physiol.* 161 1189–1202. 10.1016/j.jplph.2004.01.013 15602811

[B71] Rikin A. Richmond A. E. (1976). Amelioration of chilling injuries in cucumber seedlings by abscisic acid. *Plant Physiol.* 38 95–97. 10.1111/j.1399-3054.1976.tb04865.x

[B72] Rizwan M. Ali S. Abbas F. Adrees M. Zia-ur-Rehman M. Gill R. A. (2017). “Role of organic and inorganic amendments in alleviating heavy metal stress in oil seed crops,” in *Oil Seed Crops: Yield and Adaptations Under Environmental Stress*, ed. Ahmad P. (Hoboken, NJ: John Wiley & Sons), 224–235.

[B73] Roychoudhury A. Basu S. (2012). “Ascorbate-glutathione and plant tolerance to various abiotic stresses,” in *Oxidative Stress in Plants Causes, Consequences and Tolerance*, eds Anjum N. A. Umar S. Ahmad A. (New Delhi: IK International Publishing House Pvt. Ltd), 177–258.

[B74] Roychoudhury A. Paul S. Basu S. (2013). Cross-talk between abscisic acid-dependent and abscisic acid-independent pathways during abiotic stress. *Plant Cell Rep.* 32 985–1006. 10.1007/s00299-013-1414-5 23508256

[B75] Safrany J. Haasz V. Mate Z. Ciolfi A. Feher B. Oravecz A. (2008). Identification of a novel cis-regulatory element for UV-B-induced transcription in *Arabidopsis*. *Plant J.* 54 402–414. 10.1111/j.1365-313X.2008.03435.x 18266923

[B76] Sakamoto H. Matsuda O. Iba K. (2008). ITN1 a novel gene encoding an ankyrin-repeat protein that affects the ABA-mediated production of reactive oxygen species and is involved in salt-stress tolerance in *Arabidopsis thaliana*. *Plant J.* 56 411–422. 10.1111/j.1365-313X.2008.03614.x 18643991

[B77] Sangtarash M. H. Qaderi M. M. Chinnappa C. C. Reid D. M. (2009). Differential sensitivity of canola (*Brassica napus*) seedlings to ultraviolet-B radiation, water stress and abscisic acid. *Environ. Exp. Bot.* 66 212–219. 10.1016/j.envexpbot.2009.03.004

[B78] Schwartz S. H. Qin X. Zeevaart J. A. (2003). Elucidation of the indirect pathway of abscisic acid biosynthesis by mutants, genes, and enzymes. *Plant Physiol.* 131 1591–1601. 10.1104/pp.102.017921 12692318 PMC1540303

[B79] Sharp R. E. Wu Y. Voetberg G. S. Saab I. N. LeNoble M. E. (1994). Confirmation that abscisic acid accumulation is required for maize primary root elongation at low water potentials. *J. Exp. Bot.* 45 1743–1751.

[B80] Siedlecka A. Baszynski T. (1993). Inhibition of electron flow around photosystem I in chloroplasts of cadmium-treated maize plants in due to cadmium-induced iron deficiency. *Physiol. Plant.* 87 199–202. 10.1111/j.1399-3054.1993.tb00142.x

[B81] Singh S. Srivastava P. K. Kumar D. Tripathi D. K. Chauhan D. K. Prasad S. M. (2015). Morpho-anatomical and biochemical adapting strategies of maize (*Zea mays* L.) seedlings against lead and chromium stresses. *Biocatal. Agric. Biotechnol.* 4 286–295. 10.1016/j.bcab.2015.03.004

[B82] Singh S. Tripathi D. K. Singh S. Sharma S. Dubey N. K. Chauhan D. K. (2017). Toxicity of aluminium on various levels of plant cells and organism: a review. *Environ. Exp. Bot.* (in press). 10.1016/j.envexpbot.2017.01.005

[B83] Srivastava S. Srivastava A. K. Sablok G. Deshpande T. U. Suprasanna P. (2015). Transcriptomics profiling of Indian mustard (*Brassica juncea*) under arsenate stress identifies key candidate genes and regulatory pathways. *Front. Plant Sci.* 6:646 10.3389/fpls.2015.00646 PMC4541038 26347763

[B84] Staneloni J. R. Batiller-Rodriguez M. J. Casal J. J. (2008). Abscisic acid, high-light, and oxidative stress down-regulate a photosynthetic gene via a promoter motif not involved in phytochrome-mediated transcriptional regulation. *Mol. Plant.* 1 75–83. 10.1093/mp/ssm007 20031916

[B85] Tan B. C. Schwartz S. H. Zeevaart J. A. D. McCarty D. R. (1997). Genetic control of abscisic acid biosynthesis in maize. *Proc. Natl. Acad. Sci. U.S.A.* 94 12235–12240. 10.1073/pnas.94.22.12235 9342392 PMC23760

[B86] Thompson A. J. Jackson A. C. Parker R. A. Morpeth D. R. Burbidge A. Taylor I. B. (2000). Abscisic acid biosynthesis in tomato: regulation of zeaxanthin epoxidase and 9-cis-epoxycarotenoid dioxygenase mRNAs by light/dark cycles, water stress and abscisic acid. *Plant Mol. Biol.* 42 833–845. 10.1023/A:1006448428401 10890531

[B87] Todoroki Y. Tanaka T. Kisamori M. Hirai N. (2001). 3’-Azidoabscisic acid as a photo affinity reagent for abscisic acid binding proteins. *Bioorg. Med. Chem. Lett.* 11 2381–2384. 10.1016/S0960-894X(01)00431-0 11527736

[B88] Tossi V. Lamattina L. Cassia R. (2009). An increase in the concentration of abscisic acid is critical for nitric oxide-mediated plant adaptive responses to UV-B irradiation. *New Phytol.* 181 871–879. 10.1111/j.1469-8137.2008.02722.x 19140950

[B89] Trewavas A. J. Jones H. G. (1991). “An assessment of the role of ABA in plant development,” in *Abscisic Acid: Physiology and Biochemistry*, eds Davies W. J. Jones H. G. (Oxford: Bios Scientific Publishers), 169–188.

[B90] Tripathi D. K. Bashri G. Shweta Singh S. Ahmad P. Singh V. P., (2017a). “Efficacy of silicon against aluminum toxicity in plants: an overview,” in *Silicon in Plants: Advances and Future Prospects* Vol. 1 eds Tripathi D. K. Singh V. P. Ahmad P. (Boca Raton, FL: CRC Press), 355–366.

[B91] Tripathi D. K. Shweta Singh S. Yadav V. Arif N. Singh S., (2017c). “Silicon: a potential element to combat adverse impact of UV-B in plants,” in *UV-B Radiation: From Environmental Stressor to Regulator of Plant Growth* Vol. 1 eds Vijay P. S. Samiksha S. Sheo M. P. Parul P. (Hoboken, NJ: John Wiley & Sons), 175–195.

[B92] Tripathi D. K. Singh S. Singh S. Chauhan D. K. Dubey N. K. Prasad R. (2016b). “Silicon as a beneficial element to combat the adverse effect of drought in agricultural crops,” in *Water Stress and Crop Plants: A Sustainable Approach*, ed. Ahmad P. (Hoboken, NJ: John Wiley & Sons, Ltd.), 682–694.

[B93] Tripathi D. K. Singh S. Singh V. P. Prasad S. M. Dubey N. K. Chauhan D. K. (2017b). Silicon nanoparticles more effectively alleviated UV-B stress than silicon in wheat (*Triticum aestivum*) seedlings. *Plant Physiol. Biochem.* 110 70–81. 10.1016/j.plaphy.2016.06.026 27470120

[B94] Tripathi D. K. Singh V. P. Kumar D. Chauhan D. K. (2012). Rice seedlings under cadmium stress: effect of silicon on growth, cadmium uptake, oxidative stress, antioxidant capacity and root and leaf structures. *Chem. Ecol.* 28 281–291. 10.1080/02757540.2011.644789

[B95] Tripathi D. K. Singh V. P. Prasad S. M. Dubey N. K. Chauhan D. K. Rai A. K. (2016a). LIB spectroscopic and biochemical analysis to characterize lead toxicity alleviative nature of silicon in wheat (*Triticum aestivum* L.) seedlings. *J. Photochem. Photobiol. B Biol.* 154 89–98. 10.1016/j.jphotobiol.2015.11.008 26700425

[B96] Tuteja N. (2007). Abscisic acid and abiotic stress signaling. *Plant Signal. Behav.* 2 135–138. 10.4161/psb.2.3.4156 19516981 PMC2634038

[B97] Wang J. Lin L. Luo L. Liao M. Lv X. Wang Z. (2016). The effects of abscisic acid (ABA) addition on cadmium accumulation of two ecotypes of *Solanum photeinocarpum*. *Environ. Monit. Assess.* 188 1–8. 10.1007/s10661-016-5194-6 26899030

[B98] Wani S. H. Kumar V. Shriram V. Sah S. K. (2016). Phytohormones and their metabolic engineering for abiotic stress tolerance in crop plants. *Crop J.* 4 162–176. 10.1016/j.cj.2016.01.010

[B99] Weiner J. J. Peterson F. C. Volkman B. F. Cutler S. R. (2010). Structural and functional insights into core ABA signaling. *Curr. Opin. Plant Biol.* 13 495–502. 10.1016/j.pbi.2010.09.007 20934900 PMC2971662

[B100] Wheeler S. Loveys B. Ford C. Davies C. (2009). The relationship between the expression of abscisic acid biosynthesis genes, accumulation of abscisic acid and the promotion of *Vitis vinifera* L. berry ripening by abscisic acid. *Aust. J. Grape Wine Res.* 15 195–204. 10.1111/j.1755-0238.2008.00045.x

[B101] Xiong L. Ishitani M. Lee H. Zhu J. K. (2001). The *Arabidopsis* LOS5/ABA3 locus encodes a molybdenum cofactor sulfurase and modulates cold and osmotic stress-responsive gene expression. *Plant Cell* 13 2063–2083. 10.1105/tpc.13.9.2063 11549764 PMC139452

[B102] Xiong L. Lee H. Ishitani M. Zhu J. K. (2002a). Regulation of osmotic stress responsive gene expression by the LOS6/ABA1 locus in *Arabidopsis*. *J. Biol. Chem.* 277 8588–8596. 10.1074/jbc.M109275200 11779861

[B103] Xiong L. Shumaker K. S. Zhu J.-K. (2002b). Cell signaling during cold, drought, and salt stress. *Plant Cell* 14 S165–S183. 10.1105/tpc.000596 12045276 PMC151254

[B104] Xiong L. Zhu J. K. (2003). Regulation of abscisic acid biosynthesis. *Plant Physiol.* 133 29–36. 10.1104/pp.103.025395 12970472 PMC523868

[B105] Yamaguchi-Shinozaki K. Shinozaki K. (2006). Transcriptional regulatory networks in cellular responses and tolerance to dehydration and cold stresses. *Annu. Rev. Plant Biol.* 57 781–803. 10.1146/annurev.arplant.57.032905.105444 16669782

[B106] Yoshida R. Hobo T. Ichimura K. Mizoguchi T. Takahashi F. Aronso J. (2002). ABA-activated SnRK2 protein kinase is required for dehydration stress signaling in *Arabidopsis*. *Plant Cell Physiol.* 43 1473–1483. 10.1093/pcp/pcf188 12514244

[B107] Zeevaart J. A. Creelman R. A. (1988). Metabolism and physiology of abscisic acid. *Annu. Rev. Plant Physiol Plant Mol Biol.* 39 439–473. 10.1146/annurev.arplant.39.1.439

[B108] Zhang D. (2014). *Abscisic Acid: Metabolism, Transport and Signaling.* New York, NY: Springer.

[B109] Zhang X. Ervin E. H. (2008). Impact of seaweed extract-based cytokinins and zeatin riboside on creeping bentgrass heat tolerance. *Crop Sci.* 48 364–370. 10.2135/cropsci2007.05.0262

[B110] Zhu J. K. (2002). Salt and drought stress signal transduction in plants. *Annu. Rev. Plant Biol.* 53 247–273. 10.1146/annurev.arplant.53.091401.143329 12221975 PMC3128348

